# Mind Invasion: Situated Affectivity and the Corporate Life Hack

**DOI:** 10.3389/fpsyg.2016.00266

**Published:** 2016-02-25

**Authors:** Jan Slaby

**Affiliations:** Philosophy, Free University BerlinBerlin, Germany

**Keywords:** affect, emotion, situatedness, scaffolding, mind invasion, normativity, workplace

## Abstract

In view of the philosophical problems that vex the debate on situated affectivity, it can seem wise to focus on simple cases. Accordingly, theorists often single out scenarios in which an individual employs a device in order to enhance their emotional experience, or to achieve new kinds of experience altogether, such as playing an instrument, going to the movies, or sporting a fancy handbag. I argue that this narrow focus on cases that fit a “user/resource model” tends to channel attention away from more complex and also more problematic instances of situated affectivity. Among these are scenarios in which a social domain draws individuals into certain modes of affective interaction, often by way of attunement and habituation to affective styles and interaction patterns that are normative in the domain in question. This can lead to a phenomenon that is not so much “mind extension” than “mind invasion”: affectivity is dynamically framed and modulated from without, often contrary to the prior orientations of the individuals in question. As an example, I discuss affective patterns prevalent in today's corporate workplace. I claim that workplace affect sometimes contributes to what is effectively a “hack” of employees' subjectivity.

## Introduction

Imagine you begin as an inexperienced intern in a large company. Whatever the details of your task, it is likely that your first days in the firm will be marked by experiences like the following: You find the regular employees speaking, acting, moving, and comporting themselves in ways that are unfamiliar to you in various ways. Not only will their work routines be new to you, but also their styles of interacting, of comporting themselves, of resonating affectively with one another, the ways of address, of conversing with superiors, the use of humor to begin a conversation or deflate a moment of tension, when and how to display certain feelings openly (enthusiasm maybe, or pride after an achievement), or suppressing others (no fear, no insecurities), and so on. Accordingly, much more will be required of you than learning how the regular work routines are to be performed. You will have to habituate in various informal ways as well, in order to become “one of them,” where this being “one of them” will crucially include numerous forms of affective comportment and a particular affective style.

Now imagine that, after a successful internship, you get hired as a full-time employee and after a few month or years on the job, you find yourself assigned to assist an incoming intern on her first days in the firm. Seeing the novice stagger around the office insecurely, unsure even of how to talk, how to sit, when to smile, whom (and how) to ask for advice, it occurs to you how profoundly you yourself have been inculcated into the company's specific “style of play” (a mixture of doing and being, always on display). You might recall how strange it all was to you on your first days there, and how remarkably easy it now all comes to you. On a moment's reflection, you might also begin to see how profound and encompassing the habituation in this work environment actually is. For an instant, you grasp how much goes unquestioned: the ways co-workers are expected to display energy, eagerness, and enthusiasm, how everybody is conspicuously ready to work long hours, how firmly employees have subscribed to their divisions' goals (so that it can seem that they literally suffer or rejoice in line with the current fortunes of the company), how decidedly informal, loose, and casual conversation with those at the upper echelon is supposed to go, while it leaves remarkably little room for voicing dissenting opinion, how little tolerance for time spent offline, or how hard it is to find an open ear for concerns outside of issues of immediate relevance to work, and so on. Indeed, this is an alien land for the novice, and what seems natural here might look quite unnatural—contingent, tendentious, even outrightly hostile—from the outside.

I stop this little fictional excursion into the corporate world at this point. With it, I want to point to one of those expanded zones of contemporary life in which human affectivity is profoundly framed and modulated so that the affective and emotional dispositions of an individual squarely fall in line with the interaction routines prevalent in these domains. The corporate workplace is just one exemplary area among others—a field of in-depth affective modulation. Something similar goes on in higher education, in the world of sports, in various social-web-based subcultures, in academic departments, let alone in those classic fields of existential modulation as the military, police forces, or the ever-expanding security sector, and so forth. What characterizes these social domains in general is that they both demand and effect profound shaping of personality, importantly including affectivity. Considerations of the ways these formative social domains operate, how individuals get habituated by them, how individual comportment, affective styles, and these field's process routines intermingle, are by and large absent from recent discussions of situated affectivity in philosophy. This is part of the reason for a certain one-sidedness, not to say short-sightedness, of the otherwise helpful literature on the topic. In what follows, I will take some steps toward amending this situation, first by reviewing recent work on situated affectivity, and then by sketching a constructive proposal, geared to the complexity of cases such as affect in the contemporary white-collar workplace.

The focal issue turns on widening the scope of the debate to encompass the subjectification effects of social domains. I will argue that the individual subject whose affectivity is said to be situated, embedded or even extended is herself already a complex “product” of the sustained modulation by affect-intensive social domains. Affective habituation continues throughout adult life, with potentially deep impacts on individuals who often have little choice but to let themselves be variously framed in these ways. Thus, part of my claim is that certain forms of *socially distributed affectivity*—such as the emotional style and modes of affective engagement prevalent in a corporate workplace, crucially supported and enabled by technology and other kinds of material arrangement—are *prior to* and *formative of* individual emotion repertoires and affective-bodily styles, which themselves are part and parcel of shifting structures of subjectivity.

If we change the perspective on situated affectivity in this way, a range of cases comes in view in which it might be more accurate to speak of “mind invasion” than of “mind extension” (see Protevi, 2013, ch. 5). Here, individuals do not willingly strife to enlarge their mental repertoire by coupling to external devices in order to think better or feel more (“mind extension”; see Clark, 1997; Clark and Chalmers, 1998; Menary, 2010). Rather, social domains in which specific affective modes of interaction are prevalent and functional, effectively “seek out” domain-naïve individuals in order to turn them into bona fide exponents of the domain's operative processes. These novices' minds might get “hacked,” so to speak, especially when individual inclinations or liabilities are specifically targeted so that these persons will come to feel and comport themselves seamlessly in line with domain-conducive routines, or even adjust their habits such that pursuit of the domain's objectives will seem natural to them. If this goes discernibly against these individuals' prior orientations, and if it is in the long run detrimental to their personal flourishing, we deal with a case of “mind invasion.” I will argue that several manifestations of technology-supported affective interaction in today's white-collar workplace are cases in point.

## Affectivity, affect and emotion—initial conceptual sorting

In order to prepare for the specific discussion of situated affectivity, some brief remarks on the relevant understanding of affect, affectivity, and emotion will be helpful at the outset. For one, I approach the field of affective phenomena with an inclusive orientation, taking affective phenomena to span everything from categorical emotion types with specific intentional contents (such as anger, fear, happiness or shame), via the initially nameless, pre-intentional affective dynamics of social interaction, to unspecific moods, background feelings, or affective atmospheres. Much of my specific angle of interest in this article concerns affect in the sense of intensive transpersonal dynamics that link various interactants while also anchoring them in their surroundings. This understanding of affect, prevalent especially in the field of cultural “affect studies” (e.g., Massumi, 2002; Gregg and Seigworth, 2010), can then be differentiated further into the following three closely connected subclasses:

1. Occurrent *episodes* of situated affecting and being affected (ongoing scenes of affective intra-action)
2. Individual *affective dispositions* in the sense of a person's standing capacity to affectively resonate in specific ways (see Mühlhoff, 2015); and
3. *Social-institutional patterns* of domain-inherent affectivity, for instance the particular “affective climate” or default mode(s) of affective relatedness that characterize a corporate workplace, partly in virtue of its specific design, equipmental layout, and regular operating procedures (see e.g., Gregg, 2011).

Part of the point of the following considerations is that these three dimensions are densely interrelated, notably in the outside-in direction: i.e., a particular sustained “affective arrangement” prevalent in a social domain (type 3 above) regularly evokes both occurrent scenes of affective relatedness (type 1) and thereby, over time, habituates individuals' standing capacities or dispositions to affect and be affected in specific ways (type 2). While not in the foreground of this paper, a roughly similar story might be told for categorical emotion types and individual emotion repertoires, only that more emphasis must then be placed on the intentional contents or content templates of the emotion types under consideration (see Ahmed, 2004; Wetherell, 2012).

As will become clear from the various examples discussed below, affect and emotion are never merely matters of “internal mental states,” nor just narrow ways of being affected, but usually encompass sequences of *active engagement* with the world, usually in highly social and relational ways (see Slaby and Wüschner, 2014). This alone makes them adequate targets of normative assessments, as it might be asked whether a given sequence of affective engagement properly aligns with the particular range of activities, interactions or expressions permissible (or otherwise sanctioned) in the relevant social domain. More than that, usually it can be asked whether the affective sequence in question is conducive to realizing “the point” of the domain in which (or as part of which) it unfolds, i.e., whether the affective episode is sufficiently significant to what is ultimately at issue and at stake in the domain (while bearing in mind that these domain-specific issues and stakes are themselves open to normative contestation, continuous refinement, eventual revision and even abandonment; see Rouse, 2015, ch. 10).

As these all too brief terminological and conceptual clarifications indicate affectivity, and emotion present a complex field of phenomena which is notoriously contested. Thus, no final word is ever to be expected on how best to carve up this field. Accordingly, it is advisable for scholars to selectively focus on significant aspects in precise ways and design a conceptual map that is both: sufficiently in line with key insights and developments in the broader field of affect research, but also specific enough to take up and bring into sharp relief those features of the target phenomenon that are most relevant to addressing one's particular research interest. In the present case, this means (among other things) that forms of dynamic social-relational affectivity instead of individual emotional episodes should be at the forefront of attention^1^.

## Situated affectivity: state of the art

Recent philosophical discussions of situated affectivity harbor important insights, as they have led to a fundamental perspective shift in the philosophy of emotion. Affects and emotions are no longer seen as merely a matter of organism-bound processes but are now considered to be decisively supported and, arguably, at times even partly *constituted* by structures or processes in the emoter's environment (Griffiths and Scarantino, 2009; Thompson and Stapleton, 2009; Slaby, 2014; Krueger, 2014a; Colombetti and Roberts, 2015). This idea has led to a number of fruitful developments and inspired much interdisciplinary exchange. Some recent publications have begun to further clarify the conceptual landscape in this area—for example rendering more precise the meanings of terms such as an emotion's presumed *embodiment, embeddedness*, and *extendedness* (Wilutzky et al., 2011; Stephan et al., 2014). These clarifications might have curbed the excitement of the enthusiasts somewhat, as they have set the bar quite high for attempts to establish cases of genuine affective *mind extension*. But they have not stopped the flurry of work about affect's situatedness more broadly. Several recent publications have analyzed in detail a wide range of example scenarios, assessing to what extent these deserve to be called cases of “affective scaffolding” or even of “extended emotion” (see e.g., Krueger, 2014a,b; Candiotto, 2015; Colombetti and Krueger, 2015; Carter et al., 2016 and also several contributions to this Frontiers special topic).

In the present section, I will review some key strands of this literature. I restrict my discussion to approaches that stop short of the strong thesis of “extended emotion.” While I think that the extended emotions perspective still deserves a fair run for its money (contra some of its recent critics), a less radical proposal suffices for my present purposes.

### Griffiths and scarantino's *Emotions in the wild*

The recent surge of literature on situated affectivity was kicked off by a programmatic paper entitled *Emotions in the wild* by Griffiths and Scarantino (2009). In it, the authors propose a naturalistic, social-psychology-, and evolutionary-biology-inspired account of emotions as strategic moves within social relationships that are profoundly embedded within environmental structures, both synchronically and diachronically. Besides bringing the topic of situated affectivity on the agenda and making a convincing case for their proposal, Griffiths and Scarantino deserve credit for *not* framing the topic from the outset in the terms of the extended mind debate (e.g., in the terms set down by Clark and Chalmers, 1998; see also Clark, 2008, 2009). By remaining neutral about the question of whether the environment can literally “extent”—in the sense of “become of a constitutive part of”—the individual mind, they circumvent many of the difficulties that trouble that debate. Instead, they emphasize the less ontologically committing notion of *environmental scaffolding*. An external scaffold—a concept introduced by Clark (1997) with a nod to (Vygotsky, 1986 [1962])—is any item or structure in the environment that provides reliable support for cognitive processes, so that cognitive routines will regularly exploit these structures to enhance their functionality and effectiveness. Prime examples are public language and other symbol systems and notations, but also other artifacts such as computers, calculators, or the infrastructures of communication, and likewise the devices and tricks of various “arts of memory” or related material practices of mind, reaching back into ancient history (see also Donald, 1991; Sutton, 2010). Expanding this notion of scaffolding to encompass affectivity seems like a natural move to make (Colombetti and Krueger, 2015).

What is furthermore helpful is Griffiths and Scarantino's understanding of emotions as temporally expanded *active engagements* with—instead of merely snapshot-like passive experiences of—the world, and especially the social world. A view along these lines is gaining renewed currency in recent years, long since it had been proposed by phenomenologists such as Sartre or Merleau-Ponty.^2^ This highlights the way in which affectivity might be a matter of repeated acts of *active structuring* of the environment by an agent or a group of agents, with the aim to achieve relational goals and to effect changes in the world that are conducive to the agents' favored course of future action and experience.

In line with this understanding of affectivity as active engagement, Griffiths and Scarantino take up important insights from recent work in the sociology and social psychology of emotion (especially from Parkinson et al., 2005), in which the entanglement of individual affective comportments with social practices, group processes or even culture at large is a prominent theme. The upshot that Griffiths and Scarantino's account shares with these strands of work is that individual emotions are complex strategies of relationship configuration within communal life, embedded within substantive environmental support structures. Candidate *affective* scaffolds are things as diverse as items of material culture, engrained communal interaction practices and rituals, or even parts of the institutional set-up of a society—such as property rights, laws etc. Moreover, Griffiths and Scarantino's distinction between a diachronic and synchronic dimension of these embedding structures provides a valuable methodological directive for further work: these two dimensions make for separate strands of analysis in a candidate case of situated affect, thus informing about both, direct on-going (“on-line”) environmental shaping of affective processes *and* the longer-term historical development of the support structures in question.

### Affective scaffolding and niche construction theory

Colombetti and Krueger (2015) have expanded upon the groundwork provided by Griffths and Scarantino with their own proposal on affective scaffolding, inspired by the philosopher of biology Sterelny (2010, 2012). They follow Sterelny in transposing work in evolutionary *niche construction theory* (cf. Odling-Smee et al., 2003; Odling-Smee and Laland, 2009) to human social life, then adding affectivity to the mix to enrich the picture of the socially scaffolded mind. Niche construction amounts to a more theoretically developed approach to environmental scaffolding, as the biological notion of ecological niche allows a systematic understanding of the ways in which organisms and environments come to be structurally entangled in ever more intricate ways over evolutionary time. The crucial advance of niche construction theory over earlier paradigms of adaptationism is the idea of recursive developmental interaction between organisms and their environment. Instead of a linear story of a species' stepwise adaptation to an environment considered more or less stable, niche construction approaches hold that organisms modify their habitats in various ways while their own functional designs continue to be shaped by the layout of their respective niche. Thus, we end up with complex co-construction processes of organisms and environments. An exemplary case is again language, originally a cultural technique to which present-day human nervous systems are complexly adapted so that it is pointless to ask whether language was ultimately “cultural” or “biological” (Deacon, 1997). This insight generalizes to many other human capacities if the niche construction approach is on the right track.

Colombetti and Krueger apply Sterelny's approach to affectivity. Accordingly, they discuss several cases of *affective niches*. What Colombetti and Krueger chiefly discuss under the label of an “affective niche” are subdomains of human social life; domains such as popular music, the cinema, religion, or areas of consumer culture. For instance, music and the various socio-technological arrangements that enable, frame and enhance the listening experience, is an obvious case in point: a subdomain of social life that is effective in engendering recurring affective experiences. But even a case as mundane as the mood-dependent selection of clothing—such as putting on a brightly colored attire to counter the dullness of a rainy day—comprises a candidate affective niche in these authors' liberal proposal (Colombetti and Krueger, 2015, 1163). A further, surprising case is that of a handbag and its personalized, presumably affect-inducing contents:

A handbag—including its contents—functions as a highly portable, self-styled collection of technologies specifically chosen for regulating affect: charms and tokens for good luck and peace of mind, which influence one's appraisal of, and ability to cope with, specific situations; photos, assorted mementos (such as old theatre tickets and restaurant receipts), snippets of notes, and letters from loved ones that bring about fond memories of individuals and elicit specific feelings; and small weapons or tools that affect one's awareness of one's action possibilities, which accordingly generate feelings of confidence, power, and security. (Colombetti and Krueger, 2015, 1163).

This description of the handbag as a *portable, customized* affective niche helps bring home the pervasiveness of such forms of affective scaffolding in general, both in the private spaces of the home and in the designed spaces of civilized life (cinemas, shopping malls, event arenas of sports and entertainment, modern workplaces, etc.). It also accentuates the relevance of material culture to affective scaffolding, a theme so far under-appreciated in the philosophy of emotion.

Such a broad array of examples might raise doubts: If just any odd affect-inducing object were an instance of affective scaffolding, the proposal would be vacuous. Trust, entrenchment and individualization are the criteria discussed by Colombetti and Krueger that distinguish occasional affect inducers from those sustained and regular ones that deserve to be called “affective scaffolds.” Accordingly, habitual gaming and the affective “flow” and pleasurable absorption it reliably enables is a case of affective scaffolding, likewise entertainment events and various items of interior design in their purposeful arrangement. On the other hand, waking up in a cheerful mood because the morning sun happens to color my room brightly is not, neither is the occasional smile of a fellow traveler on the tube that might cheer me up for a few moments. The affect-inducing handbag qualifies, not least because forgetting to take it along might lead to “the sensation that something is missing, that I am not complete (…) it's as if I were amputated” (Kaufmann, 2011, 157 quoted in Colombetti and Krueger, 2015, 1165).

More relevant for present purposes are cases of *interpersonal* affective scaffolding. Concerning these, Colombetti and Krueger discuss various examples where the regular company of others—such as loved ones or friends—brings about positive feelings in reliable ways that can become deeply entrenched, that are trusted and play out in personalized ways, as in certain interactional styles or repeated forms of humorous exchange. This brings into play a broad array of scenarios, from the mildly comforting feelings of familiarity we have in the presence even of co-workers or casual acquaintances to the intensive emotions and deep background feelings we experience in the presence of our dearest companions.

Colombetti and Krueger provide a broad overview, starting from early infant-caregiver affective interactions and going all the way up to the socially orchestrated material design of public spaces, such as churches. Places of worship—to stick with the authors' own example—not only function so as to reliably induce awe, solemnity or joy, but do this in ways that are subject to deliberate modification that respond to changing tastes or needs (see Colombetti and Krueger, 2015, 1172).

A central ingredient in the authors' account of affective scaffolding and niche construction is the capacity of individuals to develop, display and adjust certain “*bodily-affective styles.”* With this concept, adopted with modifications from Husserl and Merleau-Ponty, Colombetti and Krueger begin to theorize the “interfacing” or complex entanglement between individual comportment and the various affective niches that make up the social world:

Important for our purposes is the fact that one's style is not fixed; rather, we exhibit different styles in different niches. For example, contrast how one's style transforms when teaching a classroom full of undergraduates, say, with interacting with one's partner or children, meeting professional colleagues for the first time, or going out for the evening with a group of old friends. Certain styles only seem to manifest—(…)—when scaffolded by the presence of specific social groups. (Colombetti and Krueger, 2015, 1169).

This notion of affective-bodily style with its oscillation between the relative stability of habit and the relative versatility required by multiple belongings to social spheres addresses an important theoretical desideratum. Part of the point is that these bodily-affective styles are not exclusively anchored within individual comportment and expression, but that they can be shared by the members of an in-group, such as a family, a sports team or an office work group, and that something like them can inhere communal places or social domains in the form of a recurring pattern of affective interaction that is condensed into an atmosphere or emotional climate. What also comes in view here is a certain situational “porousness” of the embodied subject as it traverses between different spheres of affective belonging (see Blackman, 2012). Due to its role as a dynamic “transit zone” between individual, social group, and material niche, the notion of affective-bodily style is specifically relevant to my own proposal developed in Sections The Socially Invaded Mind I: Distributed Affectivity and The Socially Invaded Mind II: The Case of Workplace Affect below.

## Moving beyond the user/resource model

Before I sketch my own approach to situated affectivity, I will now venture into a problematic that I think is relatively widespread in the literature on situatedness, at least as a salient tendency at the visible surface of these debates. Some of the work on the situated mind and on situated affectivity is beset by a characteristic inclination: what I call the predominance of the “user/resource model.” Baseline mentality in many of the example cases under discussion is that of a fully conscious individual cognizer (“user”) who sets about pursuing a well-defined task through intentional employment of a piece of equipment or exploitation of an environmental structure (“resource”). This oft-recurring scheme—particularly prevalent in Andy Clark style extended mind theory—might be part of the reason for why proponents of situated approaches have by and large failed to acknowledge the potentially troublesome political issues that the situatedness perspective might make visible.

To get a better sense of what I mean by the dominance of the user/resource model, we have to look no further than into the many fancy formulations that Andy Clark uses to gloss the upshot of his extended mind position. Famed Alzheimer patient Otto and his notebook (Clark and Chalmers, 1998), humans as “chimps with Filofaxes” (Clark, 2002, 37), various anecdotes starring the iPhone, or all sorts of other scenarios where individuals navigate the demands of their high-octane city and work life with an array of cognitive “props and aids” such as laptops, mobile phones, or wearable tracking devices (see e.g., Clark, 1997; 2008). Obviously, Clark also discusses the embeddedness of cognitive agents in the broader environment as an enduring support structure for their mental operations; still, the chief cases are those where the intentional employment of an isolated tool by an individual agent is at the center of attention^3^.

While their theory design is different from Clark's, and presumably more conducive to a structural reading, Colombetti and Krueger also sometimes gravitate toward individual “tools for feeling” scenarios in their examples. Even when they speak of how churches changed their interior designs, decorations and patterns of ritual as an apparent example of social scaffolding, they invoke the demands of a new generation of worshippers as pushing for these transformations—i.e., user demands as dictating how religion is done in the 21st century. The famed handbag case, the ways of relying on music in order to feel better during tedious tasks such as workouts, or the affective practice of engaging one's interlocutors and friends by the well-dosed employment of humor all fit into the user/resource grid. Likewise, the relationship configuration approach favored by Griffiths and Scarantino has more than a whiff of user/resource rhetoric about it, although the authors surely do see the potential for a broader, structural application. But even where the broader milieu is explicitly acknowledged as a structural scaffold, there still seems to be an urge to foreground individual intentions and individual comportment, at least in many of the examples under discussion.

Historians of science and technology who begin to target the 1980s have linked this pattern of thinking—and the emergence of the social type “user” in general—to the often close relationships between Silicon Valley CEOs, engineers and marketers and prominent cognitive scientists and philosophers of mind (e.g., Turner, 2006; Streeter, 2011). The individualist “user/resource” model seems to be the natural way to think about human subjectivity, in the libertarian-friendly, post-1980, California style. I won't go as far as to accuse Clark and others of an outright advertising campaign for personal computing and networked lifestyles—although the concerted efforts of Edge.org and related pundit circles might give us pause (see Stadler, 2014). What I want to highlight is a certain one-sidedness, a potential blind spot in recent work on situatedness, where the individual with his or her interests, inclinations, intentions and strategies is taken for granted as a starting point that is then placed in purposeful conjunction with a technical device or an environmental structure so that an effective coupled system of “user-plus-tool” results. Modern city- and office-dwellers might like to think of themselves in this presumably emancipated way as sovereign agents in full control of their affairs. But besides a dangerous tendency to naturalize the consumer as a template for personhood, this way of thinking risks missing out on a large variety of inadvertent structuring effects that happen outside or at the fringes of our individual purview. The term “mind invasion” is intended to capture some of the ways in which it is exactly not my individual decision to employ a mind tool in the pursuit of my self-avowed goals, but rather forms of pervasive framing and molding effected by aspects of technical infrastructure and institutional realities (Protevi, 2013; see also Verbeek, 2011).

In these cases, the relevant “affective intentionality” at play in a given scenario is not the intentionality of the individuals involved, but rather that which is structurally implemented in a distributed manner in the social domain in question. The overall affective dynamics prevalent in such scenes do have a point, they might be oriented toward domain-specific operative goals, and they likely display a systematic responsiveness to domain-relevant goings-on, and this is not just a linear, bottom-up result of the affective, or emotional orientations of the individuals implicated in the domain's activities (see below for a fuller elaboration of this point).

The short-sightedness of the user/resource model is sometimes complemented by a second troublesome tendency. Like much work in the naturalistic strands of philosophy of mind and the philosophy of cognitive science, authors writing on the situatedness of the mind often seem unwilling to sufficiently distinguish between a process-oriented and a normative understanding of its subject matter (Cash, 2010; Rouse, 2015). As has been pointed out countless times at least since Frege's attacks on psychologism, mind talk is systematically ambiguous between an “operative process” and a “normative status” understanding (these terms are helpfully brought up by Rouse, 2015, ch. 1). It is clear that an approach to the human mind worth its salt must be able to address both these dimension without running them together at random (that this does not happen very often is a different story; see Brandom, 2009). The disinterest in normativity—or the hopeless presumption that the computational theory of mind has somehow solved the issue (Fodor, 2000)—has not boded well for the prospects of recognizing the deep social framing of the mind, its constitutive beholdenness to complex socio-normative patterns (be it discursive practice or institutional structures such as the law; see also Haugeland, 1998, 2002, the latter text being a direct critique of Clark's presumed norm-blindness)^4^.

These two blind spots are related. If you are inclined to understand the mind in terms of operative processes alone, it can seem natural to think of it in terms of an individual's inbuilt mental capacities being materially augmented, scaffolded, or extended by some environmental resource. Thus, you easily end up with something resembling the user/resource model. In contrast, on a view of the human mind that takes sufficient account of its normative dimension, where individual mental states are more like public moves in a rule-governed game—or like the commitments and entitlements accrued to the games' players in virtue of their moves (see Brandom, 1994)—it will be more natural to assume that complex socio-normative patterns enable and constrain individual mental states, often in ways that are *not* explicitly reflected-about—thereby transcending the scope of the user/resource model. “Mind extension” would then potentially expand to cover communal norms and social institutions, for example in the ways discussed by work on the “socially extended mind” (Gallagher and Crisafi, 2009; Cash, 2010).

This latter view will likely bring up further important questions: Which of the many structuring demands imposed by social domains are such that they deserve the participants' reflective endorsement? And which are rather such that we see them as infringing upon our freedom, as subtle (or not so subtle) hindrances to our individual or collective flourishing, so that they are in urgent need of reform or even abolishment? A related question is then this: Do those affected have, individually or collectively, in a given case of mental situatedness, enough of a say in determining the further course that the technical and institutional infrastructures will take? With this expanded orientation we end up in the realm of the political—the situatedness debate phases over into what I call a “*political* philosophy of mind” (again, with much resonance to John Protevi's important groundwork; see Protevi, 2009, 2013; see also Massumi, 2015). The question of the constitution of individual mental capacities is here inseparable from the question of a normatively adequate organization of socio-political reality at large—where “normatively adequate” here means, at the very least: worthy of reflective endorsement by those concerned. In this sense, the human mind is a political matter as much as anything (see also Rorty, 1988). For these reasons, I applaud the moderate Hegelian turn that some authors in the philosophy of cognitive science have recently pushed for (Gallagher and Crisafi, 2009; Gallagher, 2013; Merritt et al., 2013)^5^.

On a somewhat deeper level, this perspective brings into view a problematic that is capable of making the situation even more difficult, namely that there might not be fully constituted “users”—i.e., autonomous individuals—to begin with, at least in many concrete cases of mental resp. affective situatedness. Instead, the environmental resource in question—including the normative communal practices it figures in—will itself play a role in bringing about and enabling the agent, and transforming her or him in various ways. This material-discursive subject constitution or “subjectification” is not restricted to early phases of development, but it is a matter of effective framing and re-molding of subjectivity and selfhood throughout adult life. Just think of the deep impacts communication and networking technologies have had on all our lives, affecting for good attention spans, affective reward structures, relationship habits in all sorts of ways. More pervasively still, think of gendering practices that are rampant throughout the life span, affecting all areas of human conduct and being (Young, 2005). So this is another problematic tendency inherent in user/resource approaches: the tendency to start from—and assume as largely unproblematic—fully constituted human subjects that then merely come to employ this or that environmental resource. Outside of the more elaborate versions of niche construction theory, relatively little attention has been paid to the ways in which individual subjectivity is continuously shaped by environmental structures, practices, machinery, norms and institutions. This is the question of affective *subjectification*. It likewise highlights the need for a more explicitly *political* consideration of social niches, everyday practices and various lifestyle technologies. And it brings the present approach in correspondence with previous work on subjectification and the “psychic life of power” (Butler, 1997; see also Foucaultr, 1995 [1975]; Guattari, 1995; Hacking, 2002; Allen, 2008; Haslanger, 2012 among others).

## The socially invaded mind I: distributed affectivity^6^

A good way to begin thinking of instances of affectivity outside the ambit of the user/resource model and that might qualify as cases of “mind invasion” is to take a hint from the debate on *distributed cognition* (e.g., Hutchins, 1995; Tribble, 2005; Tollefsen, 2006; Sutton, 2010). This has the advantage of not restricting the ontological domain that presumably realizes a given affective state to an individual bearer. When Hutchins discusses a ship's navigational capacities as realized by the many separate contributory acts of the crew members, or when Tribble invokes the intricate stage design and material equipment of an early modern theater company to explain its capacity to hold a staggering number of plays in its active repertoire, it is clear that the overall state or capacity in question is one that can be realized only by a collective of individuals dealing in skillful and coordinated ways with the proper kinds of equipment: “As in the expert navigational cognition described by Hutchins, so in the Globe [theatre's] physical architecture, artifacts, social structure, and the characteristics of the plays themselves combine to support the collective success of the company in performance” (Sutton, 2010, 202).

In a helpful twist in the final part of their exploration of different forms the situated affectivity perspective might take, Stephan et al. (2014) apply the insights of the distributed cognition framework to affectivity. They start out by giving the surprisingly radical example, first invoked by Max Scheler, of a mother and father at their child's grave who “as a collective, share (non-metaphorically) the same pain (*Leid*)” (Stephan et al., 2014, 76). This sounds remarkably close to a case of genuinely *extended* emotion, otherwise dreaded by these authors. But let us not be held up by exegetical intricacies. Other examples discussed in the same paper are less controversial and more in line with what I am driving at here. For instance, Stephan et al. speak of affective atmospheres or the ongoing mutual shaping of affectivity during the course of a social interaction, such as in a marital argument. This is what they consider the systematic upshot:

Cases like these, where supra-individual systems are not (…) composed of an individual coupled to some technical or non-technical artifact or natural resource but by groups of interacting individuals are the best candidates for affective phenomena that cross an individual's boundaries. (…); the motivation for not restricting affective phenomena of this kind to individuals (or their brains) is that they are an essentially collective and emergent affair. First, just as a vessel is not navigated by the navigator using his shipmates as an extrabodily resource, but by the navigational crew as a whole, (…), the oppressive atmosphere that emerges when a job interview doesn't go well and becomes awkward or the contagious euphoria or panic of a crowd do not have a single individual as a bearer but are distributed over supra-individual collectives of interacting individuals (…) [T]hey are emergent in the sense that the affective states and actions of each individual member continuously and reciprocally influence each other and are themselves shaped and amplified in a top-down manner by the overall dynamics of the group as a whole. (Stephan et al., 2014, 77).

The outside-in model of situated affectivity that I favor can take this as a starting point, and then focus especially on the ways in which individual affectivity is recursively implicated within the operations of distributed patters of affect—what Stephan et al. indicate here by speaking of top-down shaping and amplifying of individual affective states by the group's overall affective dynamic. The collective euphoria, team spirit and energized motivational climate that might be prevalent in a workplace is a good example. While on the one hand realized by the many contributory affects, expressions, interactions, performances of the individual employees, supported by structuring features of the workplace such as architecture or purposefully arranged equipment, this overall affective climate at the same time exerts profound formative influences on the affective experiences and affective engagements of the individuals that regularly dwell therein. Understood in this way, such affect-intensive social domains are a good candidate to render more concrete the notions of an “affective niche” and of “affective scaffolding” as invoked by Colombetti and Krueger.

This is a pervasive feature of organized social domains in general: that they contain formative structures—such as architecture, technological, and equipmental arrangements—that enable and help sustain recurring practices, modes of interaction and relational dynamics which, taken together, “realize” or “implement” affective interaction patterns and atmospheres. Often, these distributed affective patterns are deliberately machinated so as to help the given domain reach its operative goals, whatever these may be in the case at hand—for instance profit-making in a company, winning in a sports team, or good student performances in a school class, to take some trivial examples (see **?**). The military with its drills, disciplines, rituals and command structures is another case in point (see Protevi, 2013, ch. 2), as are the countless ways in which commercial enterprises—from shopping malls to entertainment complexes to holiday resorts—generate consumption-friendly atmospheres or implement other “technologies of allure” (Thrift, 2010)^7^.

This is recursive subject-formation or “existential modulation” if anything is—individuals crucially contribute to make up the social domains they are part of, but they are themselves shaped and molded by these domains in turn. Both goes on at the same time, in a myriad of intersecting and overlapping ways—tiny contributory acts, the simultaneous giving and receiving of form, shaping and being shaped. Here applies the central message from philosophical work on performativity (e.g., Butler, 1993): The countless contributory acts which, taken together, make up human reality, and the norms, rules and standards that presumably “apply” to this human reality are not two separate spheres, ontologically distinct. It is not embodied, physical reality on the one side and abstract, ideal rules or “ideas” on the other—instead, acts and rules, instances and patterns are co-constitutive, on the same ontological plane. Norms exist only as concretely enacted and situated, while there are no acts which are outside the ambit of social rules and normative patterns. Even presumed norm violations, outliers, perversions, or innovations have sense and readability only in relation to the established normative patterns. Applied to the human individual this obviously means that there cannot be an individual outside the ambit of socio-normative frameworks; there is literally “no body” who is not constitutively framed and molded by socially prevailing rules and categories. Butler (1993) has shown this in detail for the case of gender, seconded by Iris Marion Young—among others—on the *seriality* of social categorization (see e.g., Young, 2005, ch. 1). This and related work has often been misread as poststructuralist dissolution of solid human reality, while it is in fact a thorough *materialization* of norms, ideas, and rules in the embodied concretion of interactive material-discursive practices (see Barad, 2003).

A key meta-insight that can be transferred from performativity theory to studies of affectivity is the acknowledgment that the adequate starting point for theorizing human affectivity is not the isolated individual confronting an affect-eliciting stimulus. The adequate starting point rather is complex distributed affectivity *already prevalent* in a social domain: the ongoing back and forth of affective interaction, relational dynamics, patterns of affective engagement as it has solidified over time. It would be a severe simplification—repeating the foundational myth of “abstract individualism” (see Marx, 1973 [1858]; Foucault, 1998 [1967]) —to start with the isolated individual and her presumed individual affective states, and only then consider in what ways these individual affective states might be *further* enhanced, expanded etc. by means of certain interaction patterns, social norms, tools, resources, or techniques in the environment. Instead, theorizing has to start from a much more realistic—in the sense of: real-world adequate—starting point. It is the assumption of *in-medias-res human sociality*. There is no moment in the life of a human being where it is not in formative exposure to *ongoing, already developed* social activity (which of course includes all sorts of affective interactions). Even an infant is situated in all but a context-free, a-historic constellation. An infant's sphere of belonging is inevitably one that is massively pre-arranged, full of epoch-, culture and milieu-specific habituations, discourses, objects, norms and rules, styles of interaction, and much else (see Mühlhoff, 2015).

But let us rather focus on affective interaction scenarios in adulthood. Always already, in any domain or sphere of social life whatsoever, there is the regular commerce, the established “Betrieb” of affective interaction, to use (Heidegger, 1962 [1927]) apt term for domain-specific business-as-usual. Now a novice comes in. Emotional contagion, various synchronic, mimetic responses on a basic affective-bodily level combine with explicit demands and sanctioning on part of the established domain members to ensure the newcomer is soon swayed into and attuned with the prevailing style of affective interaction, with the affective modes of being demanded and prized by the domain in question. People habituated in accordance with the affective patterns prevalent in a social domain will reinforce and sanction the novice's affectivity from the outset, both openly and in a variety of subtle, unremarkable, often pre- or subconscious ways—by way of mimics, gesture, affective-bodily styles that signal approval or disapproval, encourage or discourage, reward with warm connection or punish with subtle hostility. A seamless censoriousness inheres human affectivity, exerted constantly in all sorts of small ways. Add to it the various atmospheres, collective feeling tones and affective styles and energy levels that inhere social places and spaces—likewise helping to sway the novice over time into consonant attunement.

Often, there is deliberate pre-selection: human resource departments, recruitment offices, or admission committees come equipped with selection procedures, tests and techniques make sure that individuals with the right inclinations and also with the right liabilities and “weak spots” are put in the right places, i.e., those positions where they will likely be most efficient for the company, army division or academic department. But increasingly also, and this is not at all incidental, people get placed where they themselves will likewise tend to feel most fitting and comfortable.

The affective “life hack” is more than merely a structure that helps corporations exploit their employees. It helps them get the most out of their workers precisely by steering them with precision into spots that present positive attachment opportunities—specific affective niches, tailor-made to pre-selected personality types or employee profiles. These affective niches then stand a good chance of getting habituated to become individual existential structures, aspects of the employees' identity—self-exploitation might then unfold smoothly and with a smile (it is “the soul at work,” see Boltanski and Chiapello, 2005; Berardi, 2009).

This is why the concept of a “hack” is appropriate: Workplace affect—the moods and affective atmospheres, the affective styles of interaction, and also the ways that digital and networked technologies machinate and channel affectivity—make up complex structures of feeling that have been set up with the express purpose of facilitating allegiance to modes of comportment, even modes of being, that will in sum prove conducive to the company's goals (see Gregg, 2011). And what is more, aren't we all passionately attached to our various circles of belonging, which encompass far more than what was formerly the “personal sphere” of family, loved ones and friends? How deep are our affective bonds to our workplace, to our “team,” to our colleagues, to the various sweet machines that make our work seamless, stylish and ubiquitous, and also to the spiritual rewards we keep aspiring to—often despite better knowledge—in the course of our imagined future journeys? These attachments are complex, ambiguous, and often cruel. They are not easy to shake for the simple reason that they *are us* (Berlant, 2012).

## The socially invaded mind II: the case of workplace affect

In order to provide some more backing for these last remarks, I close with a discussion of an example for a mind-invading structure of feeling that is currently prevalent. Obviously, I can only provide a rough sketch of what is a vast landscape of phenomena. Consider what cultural theorist Melissa Gregg has called the “presence bleed” of contemporary knowledge work (aka “immaterial labor”): the tendency, decisively facilitated by interactive technologies, that work time encroaches on what formerly were off-hours—for instance, when office workers tend to be online and available for work-related communication night and day, no matter whether on weekends or during holidays (Gregg, 2011). Likewise, with the liberation from rigid regimes of workplace presence (such as the 9-to-5 workday or the five-day week), work has tended to spread into the homes—living rooms, bedrooms, kitchens—steadily eroding the bounds that once separated work time from leisure. Gregg glosses the upshot of these developments as follows:

Presence bleed explains the familiar experience whereby the location and time of work become secondary considerations faced with a “to do” list that seems forever out of control. It not only explains the sense of responsibility workers feel in making themselves ready and willing to work beyond paid hours, but also captures the feeling of anxiety that arises in jobs that have a never-ending schedule of tasks that must be fulfilled—especially as they are not enough workers to carry the load. (…) With the increased use of digital technology, workloads that may have been acceptable to begin with are show to accumulate further expectations and responsibilities that aren't being recognized—and never will be, if home-based work continues to go unremarked. The purported convenience of the technologies obscures the amount of additional work they demand (Gregg, 2011, 2).

As these considerations show, presence bleed is not a matter of technological availability and increased task regimes alone—under the surface of permanent industriousness and “always-on” availability lurk pervasive affective tendencies. In fact, a concept like “structure of feeling” (Williams, 1977) is adequate, as we deal here not merely with some affective coloring or accompaniment of certain operative processes in labor, but with a far-reaching “mental infrastructure,” with complex patterns of affect and affective relations that play important operative roles in the universe of contemporary white collar work.

Feelings of guilt, of responsibility, various fears, and anxieties are part and parcel of this near-ubiquitous constellation, but also the excitement of connection, the thrill of being part of the action as it unfolds, or the many *petit affections* that spring up in workplace contacts—contacts which, not incidentally, have been rebranded as “friends” in the online culture of social networking (Gregg, 2011, ch. 5 and 6). It is this rampant blend of feelings and affective tendencies that characterized the contemporary dispositive of white-collar work in developed societies. In the neo-Marxist parlor of Franco “Bifo” Berardi, the upshot of this reads thus:

Putting the soul to work: this is the new form of alienation. Our desiring energy is trapped in the trick of self-enterprise, our libidinal investments are regulated according to economic rules, our attention is captured in the precariousness of virtual networks: every fragment of mental activity must be transformed into capital (Berardi, 2009, 3).

If we consider the types of affects here in question, we find among them the following: fear of being overwhelmed by the mass of messages waiting after an offline period, the anxieties of disconnection, being at risk of missing relevant developments in the office. Further, we find a recurring craving for the ambivalent satisfaction that constant communication provides, but certainly also those painful emotions of searing self-beratement as guilt or shame—creeping upon one in face of potential shortcomings in fulfilling one's work demands or failure of living up to office etiquette. Heightened feelings of inadequacy—the sense of notoriously lagging behind the standards relevant in one's professional domain—are in part a structural effect of new cultures of transparency, all-out openness, of network-enabled comparisons to competitors around the globe. Or think of the pervasive forms of workplace competition brought about by advanced performance metrics as part of new regimes of data-driven management.

As a deceptively simple example, consider the way an unanswered e-mail message can weigh upon one, exerting a subtle affective pressure until one finally goes about answering. In short, there is an intricate formation of various affective tendencies, complexly knotted-up with mobile and networked technologies, with current management styles, with the symbolic meaning of productivity and laboriousness, with structural pressures in times of flexibilized and casualized labor conditions, and with deep transformations in communication and networking habits in general. While these broader transformative tendencies in the present-day knowledge economy are well-documented in recent sociological and cultural studies literature (e.g., Liu, 2004; Boltanski and Chiapello, 2005; Gill and Pratt, 2008; Ross, 2009; Gregg, 2011), philosophers of emotion have so far shown little interest into these messy feelscapes of contemporary work.

For my purposes, it suffices at this point to briefly zoom into this dense web of practices and affects in order to identify exemplary tendencies of affective “mind invasion.” Just consider again the practice of e-mail use in white-collar work—to stick with one of the presumably least remarkable examples. There is an excitement, a certain restless expectancy about possessing an active e-mail account, as all even only remotely connected participants in the information economy will readily testify. With little regard to how boring and routine the content of most messages de facto is, we tend to eagerly await them, keep checking our inboxes repeatedly, and do it on all sorts of occasion over and over again. E-mail is part of a double-edged strategic constellation in which affectivity plays a pivotal role. The excitement inherent in the communication and connectedness enabled by e-mail is the tied up with a frantic anxiety about losing track or being left out of relevant procedures at work. The inbox has become the employees prime opening into the world. The corporate office—while less and less a stable presence in physical space—takes occupancy in one's portable computer or smartphone. Shutting off the connection equals leaving the scene of action, losing track, waning rapidly from the circle of insiders—or at least, so it will seem, so it will *feel*.

In terms of situated affectivity, the affects of connectedness that mobile communication technologies engender do show a surface resemblance to the handbag case described by Colombetti and Krueger, but this parallel does not run very deep—not least because the affects of workplace connectedness are under much less individual control. While the handbag enables a collection of handpicked items of personal comfort to be reliably present when needed, ones mobile devices are buzzing with customized heteronomy, as they let the frantic halo of the information workplace seep into one's intimate sphere of existence—with little regard for individual coping strategies or the determined resolve of the well-organized.

Examples such as these provide a welcome counterpoint to the user/resource model. As we have seen, much of the situatedness debate so far foregrounds deliberate augmentations of select mental capacities—often rather unambiguous, clearly beneficial support structures and devices. But in fact, web-based communication technologies are part of a mental augmentation structure with a far more wide-ranging, complex and ambivalent character. These devices and the communicative infrastructure they belong to are double-edged in that they exert structuring pressures on everyday routines, including affective tendencies. While they enable communicative feats of various kinds and remote access to the workplace and to relevant flows of information, they establish affective habits of rampant attentiveness, lead to hectic efforts in staying tuned, kindle anxieties of disconnection, and often provide sustained access to a sphere of activity and relatedness that quickly exceeds what an individual employee can reliably process. The world of unlimited access, while empowering, also encroaches upon various spheres of existence. It compresses time and place, it spares few spans of a knowledge workers lifetime from its influence, it helps set up and enforce encompassing regimes of attention-deployment, constant readiness, availability, and enforced productivity.

All of this provides backing for the claim I developed more abstractly above: socially instituted structures of feeling, concretely realized in domain-specific ways in technological infrastructures and affective interaction routines, affective styles, and comportments of domain members, exert far-reaching structuring effects upon those that dwell in these domains. Conspicuous among them are cases where a pleasurable, joyful, or otherwise affectively rewarding experience is generated as part of routines that also work in draining, exploitative, anxiety-evoking ways. In the long run, these routines may not only exhaust the quality of life of those involved in them, but turn them into prime exponents and “willing executioners” of the very domains, practices and process modes that have effected these feats.

## Outlook: toward a political philosophy of mind

We have good reasons to consider these affect-channeling structures of workplace technology cases of “mind invasion.” Obviously, we should not take the talk of mind invasion in an overly simplistic way, as if it was clear what a *noninvaded* mind would look like, or whether there could even be such a thing. In a more general sense, the adult human mind is *structurally* invaded—this is the point of the niche construction perspective, namely, that man-made environments back-form the mental make-up (and much else) of its regular inhabitants. And it is surely difficult to clearly demarcate those mental structures, contents or processes that are in a specific way privileged as the “true self” or the kernel of the autonomous person, from those structures, contents, or processes that are recognizable as alien impositions from without. It can be hard to tell, in a given case, what deserves my wholehearted endorsement—and what is an unwanted mind invader instead.

The human mind is always inevitably a partial crystallization of its ambient culture—it often reflects more than consciously adopts the templates, contents, and habitual styles prevalent in its formative surround. We have little choice than to become exponents, more or less typical exemplars of our ambient culture, in ways we are hardly conscious of (see e.g., Rorty, 1988). Still, we can—by means of careful probing and analysis—distinguish enabling from disabling social structures, we can assess to what extent social domains work toward setting up mental patterns that are in the long run empowering, conducive to individual and collective flourishing, or whether they are rather creating unhealthy dependencies, tie us to oppressive routines, sustain inequality, destroy communal bonds or lead to affective, and other mental habits that are detrimental to us or our kin (see also Honneth, 2012, esp. ch. 9 and 10).

In effect, we are here led to acknowledging the deeply political nature of situated affectivity and on the politics of the so-called 4EA perspective—the embodied, embedded, enactive, extended, and affective mind—more generally (see Protevi, 2009, 2013). As the examples I have discussed above make clear, not all social domains habituate their participants' affectivity in ways we can or should approve of. A sufficiently rich understanding of situated affectivity is needed in order to make these problematic constellations visible and to help those concerned see beyond the surface of positive attachment relations to their troubling backsides. This issue is so vexing because the very subjects whose evaluative outlooks are needed to make these critical assessments are themselves the targets—and ultimately, the “products”—of these formative influences. Affective attachments to and within complex social domains are a crucial dimension among those factors that constitute us as persons. Accordingly, it might seem questionable whether there can even be so much as a space for critical assessment of those very domains, practices and routines which have brought about our subjective and evaluative perspectives in the first place. How can there be enough critical distance when it is in fact the case that, plainly, *we are* our sustained affective attachments (Berlant, 2012; see also Butler, 1997, 2015)?

To address this challenge, I think a viable “political philosophy of mind” needs to join forces with those working on affect in cultural studies (see also **?**): With their careful case studies of segments of everyday life, these scholars have pioneered a mode of critical analysis that is finely attuned to the intricacies of domain-specific affective modulation (e.g., Stewart, 2007; Ahmed, 2010; Gregg, 2011; Berlant, 2012; Blackman, 2012; Cvetkovitch, 2012). The sustained formative effects of socio-political reality on individual frames of mind are studied from the vantage point of an “enlightened involvement.” Only as those directly concerned and involved—which ultimately includes all of us—can we hope to wrestle reflective insights from our ongoing entanglements with social structures of feeling. This requires a stance of sympathetic self-criticism that enables the careful—and potentially quite painful—probing and questioning of the very attachments, affective ties, and relational constellations that make us who we are.

Political philosophical of mind is impossible without the acknowledgment that even our dearest attachments and emotional habits are as much part of the problem that they might be—or might be turned into—a solution: into what assists us in “making it through the day” (Gregg, 2011, 88), but also what might eventually open up avenues for social transformation. Recent work on affect in cultural studies—for example by Lauren Berlant, Sara Ahmed, Lisa Blackman, or Melissa Gregg—can inspire and aid a sensitive philosophy of mind toward such a broadened, densely situated critical perspective. In this paper, I have attempted to get this approach off the ground by sketching a more complex understanding of situated affectivity that allows us to bring the problem of affective “mind invasion” on the philosophical agenda.

## Author contributions

The author confirms being the sole contributor of this work and approved it for publication.

## Funding

Work on this paper was supported by the German Research Foundation (DFG), as part of the Collaborative Research Center Affective Societies (SFB 1171) at Free University Berlin.

### Conflict of interest statement

The author declares that the research was conducted in the absence of any commercial or financial relationships that could be construed as a potential conflict of interest.

## Footnotes

^1^With this foregrounding of non-categorical, dynamic, relational instances of *affect* as opposed to categorical types of *emotion*, my approach overlaps substantively with the field of cultural affect studies, as it likewise emphasizes the often pre-reflexive and not-yet-individualized and not-yet-classified intra-active processes between bodies of various kinds (see Blackman, 2012 for a genealogical tracing of this line of work, and on its sometimes conflict-ridden relation to more mainstream strands of emotion theory; see also Wetherell, 2012).

^2^See Slaby and Wüschner (2014) and Wilutzky (2015) for fresh takes on the agentive nature of emotion and affectivity.

^3^Those approaches to the extended mind that focus not on considerations of *parity* (Clark and Chalmers, 1998), but instead on considerations of *complementarity* (e.g., Menary, 2007) are less prone to succumb to simplistic user/resource thinking. Complementarity approaches hold that cognitive agent and mind-extending machinery, once brought together in the relevant way, do not continue as separate existences but form a single integrated system. Accordingly, as long as this new system operates, it is not a user going about her business with a suitable tool, but rather a whole new “agent” or assemblage with a different range of operational capacities. As I have argued elsewhere, there are good reasons to free the extended mind approach from strict allegiance to the parity principle, and for pushing it toward complementarity instead (Slaby, 2014). This aligns with the following considerations in the main text, which mainly discuss cases of affective subjectification.

^4^I can only briefly highlight the normativity issue here, stopping well short of a theoretical proposal. A possible step in this could be to distinguish between the prime philosophical sense of normativity (as prevalent in the broadly Kantian tradition to which both Frege and Brandom belong) and the other, negatively tainted employment of the term, where it is a matter of socially enforced *normalization* rather than rational or moral validity (roughly, the Nietzsche-Freud-Foucault trajectory). However, in line with both Haugeland (1998,2002) and Rouse (2015) I deem it appropriate to hold these strands closely together; both authors work toward views that attempt to reconcile the normativity and normalization perspectives in a spirit of naturalism—what arguably had also been part of Heidegger's project in *Being and Time* (1962).

^5^Of course, this might also inspire a consultation of the more immediate descendants of a post-Hegelian critical perspective on subject constitution, such as present-day exponents of Frankfurt-school critical theory, in particular their work on “social pathologies” and the “paradoxes” inherent in currently salient capitalist modes of subjectification. See e.g., Hartmann and Honneth (2006), Honneth (2012).

^6^I have borrowed the phrase “socially invaded mind” and thus also the shorter gloss “mind invasion” from John (Protevi, 2013), whose important groundwork taught me much about how to think productively about socially machinated affect.

^7^Some clarification is in order on my employment of the concept “social domain,” as it plays a crucial role in the present account. It is difficult define and demarcate social domains in a precise manner, as there are so many different varieties of them—large and small, official and informal, persistent or transient, materially circumscribed or just institutionally demarcated, in all sorts of different ways. Minimally, there has to be some purposeful organization, temporal sustenance and discernible boundary, however fuzzy and shifting, between inside and outside to a bundle of social interactions (resp. practices), in order for the domain concept to validly apply. I am using the term roughly in line with what Wittgenstein-inspired practice theorist Ted Schatzki calls “social formation” (Schatzki, 1996, 199–201). Schatzki also speaks of “integrative practices” (1996: 98–108) in the settings and places these practices are both located in and help constitute. Importantly, domains and practices—and accordingly, also affective interaction patterns—are co-constitutively interrelated so that we cannot simply assign some spatial or material setting to *pre-existing* practices or affective interactions. A crucial source in the background is Heidegger, in particular his explication of “existential spatiality” (Heidegger, 1962 [1927], §§23, 24).
